# Health-Related Quality of Life in Parkinson disease: Correlation between Health Utilities Index III and Unified Parkinson's Disease Rating Scale (UPDRS) in U.S. male veterans

**DOI:** 10.1186/1477-7525-8-91

**Published:** 2010-08-30

**Authors:** Galit Kleiner-Fisman, Matthew B Stern, David N Fisman

**Affiliations:** 1 Department of Neurology, Baycrest Geriatric Hospital, 3560 Bathurst Street, Toronto, Ontario, M6A 2E1, Canada; 2 Parkinson Disease Research Education and Clinical Center (PADRECC), Philadelphia VA Medical Center, 3900 Woodland Ave, Philadelphia, PA 19104, USA; 3 Division of Epidemiology, Dalla Lana School of Public Health, University of Toronto, 155 College Street, Toronto, ON, M5T 3M7, Canada

## Abstract

**Objective:**

To apply a scaled, preference-based measure to the evaluation of health-related quality of life (HRQoL) in Parkinson's disease (PD); to evaluate the relationship between disease-specific rating scales and estimated HRQoL; and to identify predictors of diminished HRQoL.

**Background:**

Scaled, preference-based measures of HRQoL ("utilities") serve as indices of impact of disease, and can be used to generate quality-adjusted estimates of survival for health-economic evaluations. Evaluation of utilities for PD and their correlation with standard rating scales have been limited.

**Methods:**

Utilities were generated using the Health Utilities Index Mark III (HUI-III) on consecutive patients attending a PD Clinic between October 2003 and June 2006. Disease severity, medical, surgical (subthalamic nucleus deep brain stimulation (STN-DBS)), and demographic information were used as model covariates. Predictors of HUI-III utility scores were evaluated using the Wilxocon rank-sum test and linear regression models.

**Results:**

68 men with a diagnosis of PD and a mean age of 74.0 (SD 7.4) were included in the data analysis. Mean HUI-III utility at first visit was 0.45 (SD 0.33). In multivariable models, UPDRS-II score (r^2 ^= 0.56, P < 0.001) was highly predictive of HRQoL. UPDRS-III was a weaker, but still significant, predictor of utility scores, even after adjustment for UPDRS-II (P = 0.01).

**Conclusions:**

Poor self-care in PD reflected by worsening UPDRS-II scores is strongly correlated with low generic HRQoL. HUI-III-based health utilities display convergent validity with the UPDRS-II. These findings highlight the importance of measures of independence as determinants of HRQoL in PD, and will facilitate the utilization of existing UPDRS data into economic analyses of PD therapies.

## Introduction

Parkinson's disease (PD) is a chronic neurodegenerative illness that results from progressive cell death affecting movement, mood, cognition and autonomic function [1]. The prevalence of PD is approximately 1% among those aged greater than 65 [2]. A 2005 estimate placed the number of individuals aged over 50 living with PD in the world's ten most populous countries at 4.1-4.6 million, with projected increases to 8.7-9.3 million by 2030 [3].

The precise effect of optimal PD treatment on life expectancy is unclear, but living with this chronic degenerative illness is thought to have a profound negative impact on health-related quality of life (HRQoL) due to both disease manifestations, and the adverse effects of medical and surgical management strategies [4-9]. As such, the public health burden of PD is significant and increasing, and ways of assessing the impact of therapeutic interventions on HRQoL are needed for optimal patient care and for allocation of scarce healthcare resources [10].

The Unified Parkinson Disease Rating Scale (UPDRS) consists of assessments in 4 domains including, mood and cognition (UPDRS I), activities of daily living (UPDRS II), motor symptom severity (UPDRS III) and complications of treatment (UPDRS IV) [11]; it is the standard and most commonly used rating scale for disease severity in PD, however, it does not explicitly capture HRQoL, and has not been validated for this purpose. Generic measures of HRQoL take into account such dimensions as functional capacity, emotional well being, and role function that may not be adequately captured by disease rating scales [12]. Furthermore, generic HRQoL instruments allow comparison of health-related quality of life across different disease states. While questionnaires for evaluation of HRQoL in PD (such as the PD-39 and Parkinson's Disease Quality of Life instruments [13] have been developed, these instruments are neither scaled nor preference-based. Scaled, preference-based HRQoL measures ("health utilities") can also be used to "quality-adjust" survival estimates, and are easily incorporated into health economic analysis of medical interventions [14].

Given the increasing awareness of HRQoL as an important end-point that may not correlate directly with physical disability, there has been a growing literature documenting the predictors of low HRQoL in PD [15-17]. However, there have been relatively few attempts to quantify health utilities [9], or to evaluate the relationship between utilities and PD-specific rating scales such as the UPDRS. As there is a large volume of intervention-specific data already accumulated using the standard UPDRS, and very limited amount of data captured regarding HRQoL, a means of translating UPDRS data into HRQoL would be extremely valuable and would permit cost-utility analysis of interventions incorporating data that have already been collected. We sought to measure both disease severity and health utilities in PD, through parallel application of disease specific rating scales and the Health Utilities Index-III (HUI-III), an easy to use, well-validated instrument useful for approximation of scaled, preference-based health utility measures of HRQoL. Our objectives were to evaluate the relationship between disease severity (as measured by standard rating scales), and estimated health-related quality of life in individuals with PD, and to identify predictors of diminished HRQoL.

## Methods

### Subjects

The study population consisted of individuals attending the Philadelphia Veterans Administration Parkinson's Disease Research, Education and Clinical Center (PADRECC) between October 2003 and June 2006 with an ICD-9 diagnosis of Parkinsonism or PD. The PADRECC is a multidisciplinary center providing subspecialty care to veterans with PD and other movement disorders and serves a catchments area that covers Pennsylvania, New England and the Mid-Atlantic States. The population of veterans receiving medical care through the Veterans Administration healthcare system in this area is 998,061, of whom approximately 5303 have diagnosed PD. Individuals from this cohort are referred to PADRECC for expert guidance on disease management. Charts of all patients attending the PADRECC during the study period were reviewed. As this was a longitudinal prospective cohort study with respect to the outcome of interest (HUI-III), only individuals with at least 2 completed HUI-III questionnaires (from 2 separate visits) were eligible for inclusion. Review of the diagnosis of parkinsonism was further scrutinized and only individuals fulfilling United Kingdom Brain Bank Criteria [18] for idiopathic PD (IPD) were included in the database. Information abstracted from the medical record included age of disease onset, disease duration, gender, marital status, living arrangements, and level of education, as well as information on co-morbid medical conditions that might reduce health-related quality of life [19], including diabetes mellitus [20], coronary artery disease [21], stroke [22] and arthritis [23]. PD severity was assessed using UPDRS ADL and motor sub-scores (UPDRS II and III) [11], the Hoehn and Yahr Score (H+Y) [24], and the Schwab and England Disability Score (S+E) [25]. Assessments were performed in the "on" state. Medication dosages, presence of motor fluctuations and dyskinesia, surgical intervention (STN-DBS), and non-motor symptoms including depression, dementia, psychosis, drooling, urinary dysfunction and constipation were also abstracted from the records. Depression, dementia and psychosis were deemed to be present if explicitly documented in the chart. Additionally, these diagnoses were presumed if anti-depressants, neuroleptics, cholinesterase inhibitors, or other cognitive enhancing drugs were prescribed. The study was approved by the Institutional Review Board of the Philadelphia VA Hospital. All analyses were performed using Intercooled Stata Version 10.0 (Stata Corporation, College Station, TX).

### Measurement of HRQoL

Health utilities are scaled, preference-based generic measures of health-related quality of life that lie on a zero-to-one scale, with a utility of 1 equivalent to perfect health, and 0, equivalent to death. (Scores less than 0 are possible, and could be interpreted as health states less desirable than death). While utilities can be elicited using "standard-gamble" or "time-tradeoff" methods, these are intellectually rigorous, and may be upsetting to study subjects [14]. The use of a "health index" approach has several advantages with respect to elicitation of health utilities, including ease of administration, avoidance of distressing scenarios, and the potential for self-administration by subjects [26]. The HUI-III is an easy to use, well-validated instrument useful for approximation of scaled, preference-based health utility measures of HRQoL. In the HUI-III, rankings on eight health domains (including cognition, vision, hearing, speech, ambulation, dexterity, emotion, and pain) are transformed using a function that maps these domains onto utility scores that reflect community preferences [27]. HUI-III data were obtained from medical records, as the instrument was incorporated into the standard clinic intake form in October 2003.

### Statistical Analyses

We performed both cross-sectional analyses on baseline data collected for the study cohort, and longitudinal analyses in which we evaluated change in utility scores over time. Baseline HUI-III-based utility scores were evaluated for the cohort as a whole using descriptive statistics. The relationships between UPDRS scores and raw and log-transformed HUI-III utilities were assessed graphically. We evaluated the association between baseline patient characteristics (including PD severity) and baseline HUI-III scores through construction of bi-variable least-squares regression models, with standard errors adjusted to account for multiple measurements on some study subjects. Characteristics that were associated with HUI-III scores at the *P *< 0.15 level were considered candidate covariates in multivariable regression models. Multivariable models were constructed using a stepwise selection algorithm, with covariates retained for *P *< 0.15 [28]. We created a multivariable model ("Model 1") in which the UPDRS II and III sub-scores were used as candidate covariates, but also created an alternate model in which components of UPDRS II and III, rather than overall scores, were included individually as covariates. The balance between model precision and parsimoniousness was assessed using Akaike's information criterion (AIC) [29]. Interaction between model covariates was explored using multiplicative interaction terms.

Longitudinal changes over time in HUI-III scores, and UPDRS scores, were evaluated using repeated-measures ANOVA. For the subset of individuals (N = 20) for whom repeated HUI-III *and *UPDRS scores were available, we further explored the relationship between change in HUI-III scores and UPRDS III scores using the approach of Fitzpatrick et al. [4], with calculation of changes between first and last measurements for both scores, and rescaling of scores by dividing by standard deviations in scores. Correlation between changes were evaluated through calculation of Spearman correlation coefficients. We also created multivariable regression models to evaluate predictors of change in HUI-III-based utilities between first and last evaluation.

## Results

### Study Population

We screened 156 consecutive patients assessed for parkinsonism in our clinic over the study period. Of these 88 (57%) had more than 1 evaluation of health-related quality of life, and so were included in the study. Of these individuals, 20 had parkinsonism but did not meet Brain Bank criteria for PD; among excluded individuals six were diagnosed with likely vascular parkinsonism; eight were excluded based on atypical features not suggestive of idiopathic Parkinson's disease, two each were excluded based on diagnoses of multisystem atrophy and suspected diffuse Lewy body dementia, and one each was excluded based on diagnoses of fronto-temporal dementia, and progressive supranuclear palsy.

Baseline patient characteristics are outlined in Additional File 1: Table S1. All 68 included individuals were male. Of these, all had at least 2 visits, 28 had 3 visits and 3 had 4 visits during the study period. Median follow-up time was 210 days (interquartile range 159-546). The mean age at first evaluation was 73.6 years. The majority of patients lived at home either independently or with family assistance. Most patients had at least a high school education; 18% achieved grade school or less.

Comorbid medical conditions identified in the cohort included coronary artery disease, stroke, arthritis and diabetes mellitus. On average subjects had disease duration of 8 years at the time of the first recorded visit, with moderate disease severity (reflected by an average UPDRS III score of 30 and H+Y score of 2.8). Mean dosage of anti-parkinsonian medications, expressed as levodopa equivalent dose (LED) [30], was 719 mg/day. Motor fluctuations and dyskinesia were relatively uncommon; there was a high prevalence of non-motor symptoms of depression, urinary frequency and urgency, and constipation. Cognitive impairment was present in approximately 15% of patients at first visit; the mean baseline mini-mental status exam score in the cohort was 27.5 (SD = 3.0).

### Baseline Health-Related Quality of Life

**Figure 1:**
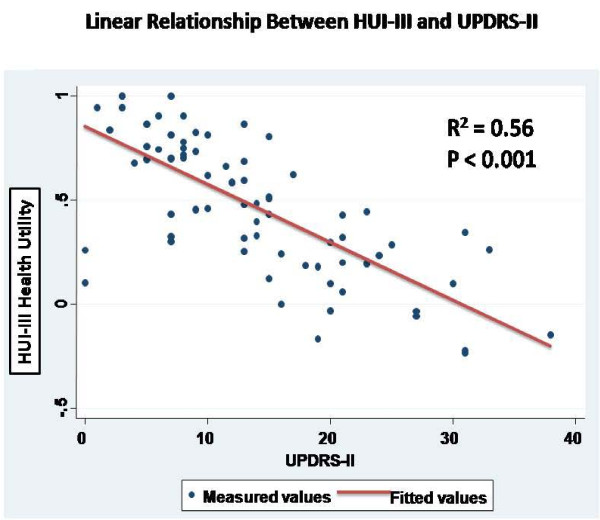
**Relationship between UPDRS II scores (X-axis) and HUI-III utlity estimates (Y-axis) showing approximately linear realtionship**.

The average value for baseline HUI-derived utility weights was 0.42 (range -0.15 to 1.0). In univariable regression models, stroke was significantly associated with reduced HUI-derived utility weights; borderline significant associations were seen with diabetes and marital status (Additional file 1; Table S1). However, several disease characteristics were found to be predictive of low baseline HRQoL, including disease duration, disease severity as reflected by H+Y scores, S+E scores, and UPDRS II and III scores (Figure 1). Consistent with this, collinear variables such as individual UPDRS motor sub-scores of bradykinesia, rigidity, and summed axial sub-scores (PIGD and ADL-axial) also predicted lower utility scores.

Motor fluctuations, though mild in the few patients that endorsed them, were correlated with low baseline quality of life. Non-motor symptoms of dementia, depression, psychosis, urinary dysfunction, and drooling were all significantly associated with decreased HRQoL in univariable analysis.

### Multivariable Regression

We created two best-fit multivariable regression models for prediction of HUI-III utilities based on UPDRS scores, sub-scores, and other patient characteristics (Table 1). The first model ("Model 1") used UPDRS-II and -III scores as candidate covariates, while "Model 2" used UPDRS sub-scores (tremor, bradykinesia, rigidity, PIGD, ADL-axial) as candidate covariates. In Model 1, both UPDRS-II scores and S+E scores were independent predictors of HRQoL; UPDRS-III was no longer significantly associated with HRQoL after controlling for UPDRS-II and S+E scores.

**Table 1:** Best Fit Multivariable Regression Models with UPDRS Summary Scores as Candidate Variables (Model 1) and UPDRS Component Sub-Scores as Candidate Variables (Model 2)

	Multivariable Model 1 r^2 ^= 0.69, AIC = -21.4			Multivariable Model 2 R^2 ^= 0.76, AIC = -33.1		
**Predictor**	**Coefficient**	**95% CI**	***P*-value**	**Coefficient**	**95% CI**	***P*-value**

**Intercept**	0.25	---	---	0.17	---	**---**
**UPDRS II**	-.015	-0.024 to -0.005	0.003	---	---	---
**Axial Subscore**	---	---	---	-0.030	-0.043 to -0.018	<0.001
**Schwab and England Score**	0.006	0.002 to 0.010	0.003	0.005	0.003 to 0.008	<0.001
**Pharmacotherapy for Dementia**	-0.21	-0.37 to -0.04	0.02	---	---	---
**Duration of disease**	---	---	---	0.016	0.004 to 0.027	0.007

**Figure 2:**
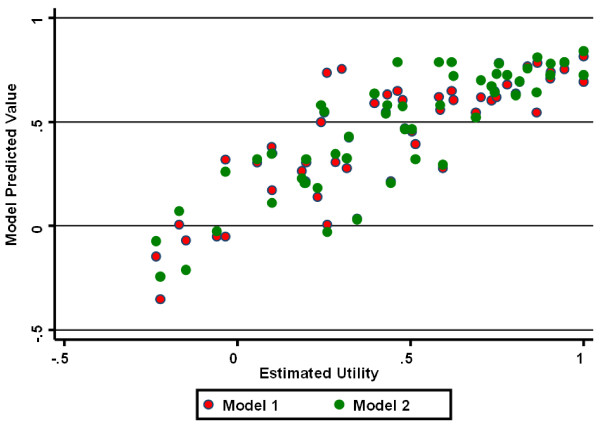
**Relationship between measured HUI-III utility estimates (X-axis) and predicted estimates (Y-axis) using multivariable model 1(red circles) and multivariable model 2 (green circles) which are described in greater detail in the text**. For both models, the relationship between observed and expected utility estimates was approximately linear.

In Model 2, UPDRS axial sub-scores (PIGD and ADL-axial) and S+E scores were independent predictors of HRQoL; increased disease duration was associated with *increased *HRQoL after adjustment for axial sub-scores and S+E scores. Both models explained a high proportion of between-subject variation in HRQoL, and both models displayed excellent predictive ability (Figure 2).

### Change Over Time

The average time interval between first and last assessment in the cohort was 6.6 months (SD 4.9). The mean reduction in HUI-III utilities between first and last assessment was 0.014 (SD 0.25); 34 individuals (50%) experienced a net reduction in utility, 33 (49%) experienced a gain in utility, and 1 (1%) had no change in health utility. When utilities were analyzed using repeated measures ANOVA, there was no reduction in utility scores with succeeding visits (P = 0.67). Significant changes were identified in Schwab and England scores (P = 0.02), but not in UPDRS-III scores (P = 0.66) or Hoehn and Yahr scores (0.11) using a similar approach. Repeated measurements of UPDRS-II scores were obtained in only 20 of 68 subjects; there was no significant change over time in these scores (0.50).

Notwithstanding the small number of individuals with both repeated HUI-III and UPDRS measurements, significant Spearman correlations were identified between changes in HUI-III scores (rescaled by dividing by standard deviations in changes) and rescaled change in UPDRS-III scores (rho = 0.25, P = 0.045), Schwab and England scores (rho = -0.38, P = 0.003), and Hoehn and Yahr scores (rho = 0.31, P = 0.017). The largest correlation coefficient was observed for rescaled change in UPDRS-II scores, though because of the small numbers of individuals with repeated UPDRS-II measurement this was not statistically significant (rho = 0.39, P = 0.093). In a multivariable regression model, changes in HUI-III utilities were predicted only by changes in UPDRS-III scores (change per unit increase in UPDRS-III score -0.009, 95% CI -0.016 to -0.002) and time between first and last evaluation (change per week -0.017, 95% CI -0.028 to -0.006).

## Discussion

Although Parkinson's disease is most prominently identified with physical symptoms such as tremors and akinesia, this disease has a substantial impact beyond motor impairment and physical disability with an on overall reduction in all health-related quality of life dimensions including social and emotional well-being. To date, the relatively limited application of existing tools for the measurement of health-related quality of life (HRQoL) has made it difficult to compare the loss of HRQoL in PD to that experienced by individuals with other chronic conditions [9]. Using a health utilities "index" approach we found a substantial reduction in HRQoL in a cohort of individuals attending a PD specialty clinic, similar to other reports [16,31-34]. However, we also found that diminished HRQoL as measured by changes in health utilities was closely correlated with changes in scores on a PD-specific disease severity measure, the UPDRS.

### HUI and UPDRS

We are aware of at least one other prior effort to map health utilities onto UPDRS scores [9]; Siderowf and colleagues identified agreement between overall UPDRS scores and the HUI-II, as well as other utility-based instruments. Our mean utility estimate (0.42) is lower than that reported by Siderowf et al. (with a mean utility of 0.74), and this may reflect the fact that our cohort was assembled at a clinic to which patients were referred due to complexities of medical management, and could also reflect a different profile of co-morbid conditions in the two populations. It may also, in part, reflect the fact that HUI-III includes domains (such as vision and hearing) that are not included in HUI-II, and which may be sources of diminished global quality of life in the age group at greatest risk of PD.

In comparison to the Siderowf study, our study further refined the relationship between health utilities and UPDRS scores. Perhaps surprisingly, we found that these reductions were most strongly correlated with the self-care component of the UPDRS (UPDRS-II), rather than the UPDRS-III motor sub-score. This finding serves as an important reminder that loss of independence may be an important source of morbidity in individuals with PD. As we demonstrated in regression analyses (Figure 1), the correlation between UPDRS-II and HUI scores was so substantial that it may be possible to generate approaches whereby existing disease-specific scores can be transformed into health utility estimates, for the purposes of comparing the health burden associated with PD to that seen in other chronic medical conditions, and in order to utilize HRQoL as the outcome of interest in economic evaluations of novel therapies for PD.

### Predictors of Low Baseline HRQoL

Other important predictors of low baseline HRQoL in this study included reductions in S+E disability scores, and higher axial sub-scores (PIGD). Though health-related quality of life and self-care ability in PD are inextricably linked to severity of motor dysfunction, the relationship between motor impairment and reduction in health-related quality of life may be complex and indirect, as demonstrated by our failure to find an independent relationship between UPDRS motor III sub-scores and HUI, after controlling for UPDRS-II and other scores. These results are consistent with previous findings that motor impairment in and of itself does not reduce health-related quality of life but the functional consequences of poor motor function including loss of self-care capabilities, inability to ambulate and loss of independence and its emotional consequences that may provide the link between physical impairment and low HRQoL [16,35].

We failed to find an association between either cognitive impairment or evidence of depression and low HRQoL, similar to one other study [15]. However this lack of association may reflect the fact that our study population was relatively intact cognitively (mean MMSE = 27.5/30). Nonetheless, it is also well-recognized that the MMSE is insensitive to capturing early cognitive decline in PD patients [36] and therefore we may not have identified individuals with subtle cognitive changes. Alternatively, it is possible that the mild cognitive changes in this cohort were insufficient to contribute substantially to low HRQoL.

Six prior longitudinal studies have evaluated HRQoL in PD. The first, based on a community-based cohort, found no relationship between any baseline clinical characteristics and reduction in HRQoL [37]. Another study [31] using both disease specific measures (PDQL and PDQ-39) and a generic utilities-based instrument (EQ-5D) did not identify change in HRQoL over time using the EQ-5 D. However, low disease-specific quality of life scores in general were predicted by depression, motor complications, cognitive impairment, and gait instability. The lack of change in the EQ-5 D was attributed to short follow-up time (12 months); the authors also postulated that the EQ-5 D was not sufficiently sensitive to pick up the subtle changes that may have occurred over only 1 year. A third study, by Fitzpatrick and colleagues [4], identified correlation between a generic HRQoL measure (SF-36) and a disease-specific HRQoL measure (the PDQ-39) (neither of them scaled nor preference-based) and also identified correlation between these measures in change over time [4], similar to the findings reported here.

Forsaa et al. [15] prospectively followed patients for 4 to 8 years, with HRQoL measured using the Nottingham Health Profile (NHP), a validated generic instrument. This study found that the greatest predictor of reduction in HRQoL was decline in physical mobility (as captured in part by worse S+E scores and higher H+Y scores), though depression and sleep disturbance were also important contributing factors; Contrary to our findings, UPDRS-II sub-score was not found to predict reduction in HRQoL.

Marras et al. also evaluated predictors of diminished HRQoL [16] using a large cohort from the DATATOP database. HRQoL was evaluated using the physical component sub-score (PCS) and mental component sub-score (MCS) of the SF-36, a generic HRQoL scale. Depression and self-rated cognitive function predicted low PCS; low MCS was predicted by older age and S+E disability scores at baseline. HRQoL and PIGD sub-scores declined in parallel over time. As in our study, these authors suggested that physical impairments associated with PD did not directly reduce health-related quality of life. Rather, lower health-related quality of life reflected diminished ability to perform ADLs, with increased dependence on others. Most recently, Brown and colleagues evaluated the relative performance of SF-36 and PD-specific quality of life instruments in predicting change in criterion indices of disease severity and quality of life (measured with a visual analogue scale); disease-specific measures outperformed generic measures in explaining variance in criterion indices, though SF-36 was more responsive to change over time [13].

### Change Over Time

Health utility estimates and most indices of PD severity were relatively stable over the course of our study, which may reflect the relatively short duration of study, and perhaps also the fact that notwithstanding the decline in status expected with a degenerative disease like PD, at least some subjects may have experienced improved health-related quality of life as a result of optimized medical management following referral to the PADRECC. Changes in utility were correlated with changes in multiple PD-specific measures, though our ability to document relationships between changes in health-related quality of life and changes in UPDRS-II scores were limited by the fact that repeated UPDRS-II scores were available in only a small subset of subjects.

### Limitations

This study had several important limitations. Our failure to identify a link between depression and low HRQoL contrasts with the results of other studies [15,38-45] and could reflect misclassification of depression, which was based on records of physician diagnosis or prescription of antidepressant medication, rather than through standardized prospective assessment. Studies that have identified associations between depression and low HRQoL have generally confirmed depression using validated mood assessment instruments. As such, our failure to find an association between depression and HRQoL in patients with PD should be interpreted with caution.

Other limitations of this study relate to the generalizability of findings in a cohort of male U.S. veterans: our findings may not be generalizable to non-veterans or to women, as they were not represented in our cohort. Previous epidemiological surveys have suggested gender differences in PD; Men have been described to have earlier symptom onset [46], increased incidence of cognitive impairment [47], increased risk of pathological gambling [48] and decreased rates of depression [49]. Women have cited greater disability and lower health-related quality of life in comparison to men with PD [50]. Finally, as discussed above, we had a limited ability to assess changes in UPDRS-II scores over time as these measurements were repeated infrequently.

## Conclusions

In conclusion, we sought to evaluate health-related quality of life in PD using a "health utilities index" approach, and to assess the relationship between health utility scores and PD severity as measured using standard disease-specific tools. In cross-sectional analyses, we identified ADL-related components of the UPDRS as most closely linked to health-related quality of life, a finding that underscores the fact that PD manifests in dimensions aside from movement and motor control. Our findings, although preliminary, may pave the way for translation of PD-specific measures of disease severity into health utility scores, particularly if our findings can be replicated and externally validated in other populations and by other investigators.

## Authors' contributions

GKF was responsible for study conception, development of the study protocol, data collection and analysis. She wrote the first draft of the manuscript and revised the manuscript for important intellectual content. MBS was responsible for study conception, contributed to the development of the study protocol, and revised the manuscript for important intellectual content. DNF contributed to development of the study protocol, and data analysis, and revised the manuscript for important intellectual content. All authors have seen and approved the final manuscript draft.

## Competing interests

The authors declare that they have no competing interests. GFK had full access to all of the data in the study and takes responsibility for the integrity of the data and the accuracy of the data analysis

## Supplementary Material

Additional file 1 Table S1: Characteristics of PD patients at the Philadelphia PADRECC at First Visit and Relationship with Health-Related Quality of Life in Univariable Regression Models Click here for file
