# Supplementary material for: Health-Related Quality of Life in Parkinson disease: Correlation between Health Utilities Index III and Unified Parkinson's Disease Rating Scale (UPDRS) in U.S. male veterans
Source: Health Qual Life Outcomes. 2010 Aug 30;8:91. doi: 10.1186/1477-7525-8-91 (PMC2939643; doi:10.1186/1477-7525-8-91)
Supplement: Additional file 1 — Table S1: Characteristics of PD patients at the Philadelphia PADRECC at First Visit and Relationship with Health-Related Quality of Life in Univariable Regression Models [file 1477-7525-8-91-S1.DOC]

**Table S1: Characteristics of PD patients at the Philadelphia PADRECC at First Visit and Relationship with Health-Related Quality of Life in Univariable Regression Models**

| **Subject Characteristic** | **Value at First Visit** | **Univariable Model Coefficient (95% CI)** | ***P*-value** |
| --- | --- | --- | --- |
| Mean age (years, (SD)) | 73.6 (7.2) | -0.005 (-0.017 to 0.007) | 0.41 |
| Male gender (N, (%)) | 68 (100.0) | --- | --- |
| Marital status* (N, (%)) | --- | -0.20 (-0.40 to -0.0009) | 0.05 |
| Married | 61 (88.2) | --- | --- |
| Divorced | 3 (4.4) | --- | --- |
| Single | 1 (1.5) | --- | --- |
| Widowed | 3 (4.4) | --- | --- |
| Education* (N, (%)) | --- | 0.055 (-0.12 to 0.23) | 0.53 |
| Grade school | 12 (17.6) | --- | --- |
| High school | 22 (32.4) | --- | --- |
| Some college | 7 (10.3) | --- | --- |
| College graduate | 13 (19.1) | --- | --- |
| Graduate school | 10 (14.7) | --- | --- |
| Unknown | 4 (5.9) | --- | --- |
| Living Arrangement* (N, (%)) |  | -0.037 (-0.093 to 0.019) | 0.19 |
| Independent | 8 (11.6) | --- | --- |
| Family assistance | 54 (79.4) | --- | --- |
| Private with nursing assistance/retirement community/assisted living | 3 (4.4) | --- | --- |
| Unknown | 3 (4.4) | --- | --- |
| Median disease duration (years, (range)) | 8 (0-32) | -.019 (-0.030 to -0.008) | 0.001 |
| Mean/median disease severity |  |  |  |
| Hoehn & Yahr score (median, (range)) | 2.5 (2-5) | -0.24 (-0.32 to -0.17) | <0.001 |
| Schwab & England score (mean, (SD)) | 74.7 (18.7) | 0.011 (0.009 to 0.014) | <0.001 |
| UPDRS II score (mean, (SD)) | 15.6 (8) | -.027 (-0.036 to -0.018) | <0.001 |
| UPDRS III score (max 108) (mean, (SD)) | 30 (12.6) | -0.015 (-0.018 to -0.011) | <0.001 |
| UPDRS motor component scores |  |  |  |
| Tremor sub-score (max 28) (median, (range)) | 2 (0-16) | 0.002 (-0.019 to 0.022) | 0.20 |
| Bradykinesia sub-score (max 32) (mean, (SD)) | 10.8 (6.0) | -0.030 (-0.037 to -0.023) | <0.001 |
| Rigidity sub-score (max 20) (mean, (SD)) | 6.2 (4.5) | -0.032 (-0.043 to -0.020) | <0.001 |
| PIGD sub-score (max 20) (mean, (SD)) | 5 (4.5) | -0.056 (-0.071 to -0.041) | <0.001 |
| ADL-Axial sub-score (max 24) (mean, (SD)) | 7.0 (4.7) | -0.049 (-0.064 to -0.034) | <0.001 |
| Complications of therapy |  |  |  |
| Motor fluctuations (proportion of day “OFF”, 0-4 scale) (median, (range)) | 0 (0-4) | -0.079 (-0.16 to -0.003) | 0.04 |
| Dyskinesia duration, disability and pain sub-score (max 12) (median, (range)) | 0 (0-5) | -0.065 (-0.13 to -0.0009) | 0.05 |
| Non-motor symptoms (N, (%)) |  |  |  |
| Urinary urgency/frequency | 43 (62.3) | -0.26 (-0.42 to -0.10) | 0.002 |
| Drooling | 32 (47.1) | -0.23 (-0.38 to -0.07) | 0.005 |
| Constipation | 28 (41.2) | -0.014 (-0.19 to 0.16) | 0.87 |
| Medication use |  |  |  |
| Levo-dopa doseage equivalent (mg, (SD)) | 719.1 (368.63) | -0.0002 (-0.0003 to -0.00005) | 0.01 |
| History of depression or current use of antidepressant medications (N, (%)) | 21 (42.7) | -0.25 (-0.40 to -0.08) | 0.002 |
| History of psychosis or current use of neuroleptic medications (N, (%)) | 7 (10.3) | -0.45 (-0.60 to -0.30) | <0.001 |
| Use cognition-enhancing medications† (N, (%)) | 10 (14.7) | -0.29 (-0.44 to -0.13) | <0.001 |
| Mini-mental status examination score (median, (range)) | 28 (18-30) | 0.040 (0.0077 to 0.072) | 0.02 |
| Medical comorbidities (N, (%)) |  |  |  |
| Coronary artery disease | 21 (30.9) | 0.029 (-0.13 to 0.19) | 0.72 |
| Stroke | 2 (3.0) | -0.44 (-0.60 to -0.28) | <0.001 |
| Diabetes mellitus | 8 (11.8) | -0.28 (-0.57 to 0.006) | 0.06 |
| Arthritis | 16 (23.3) | 0.099 (-0.11 to 0.31) | 0.35 |
| Health Utilities Index-III score (mean, (SD)) | 0.42 (0.31) | --- | --- |

* In univariable regression, evaluated relationship between married (yes/no) and HUI-III scores; college graduate (yes/no) and HUI-III scores; and living independently (yes/no) and HUI-III scores.

† Including memantine, donepezil, or rivastigmine. Tremor subscore; sum of Unified Parkinson Disease Rating Scale (UPDRS) items 20: Rest tremor and 21: Postural and action tremor of hands; Bradykinesia subscore; sum of UPDRS items 23: finger taps, 24: hand grips, 25: hand pronation/supination, 26: leg agility, Rigidity sub-score; UPDRS item 22, PIGD: postural instability & gait disturbance sub-score; sum of UPDRS items 13: Falling, 14: Freezing, 15: Walking, 29: Gait and 30: Postural stability, ADL-axial sub-score; sum of UPDRS items 5: speech, 7: swallow, 12: turning in bed, 13: falling, 14: freezing, and 15: walking.
